# Effects of Huang Bai (*Phellodendri Cortex*) on bone growth and pubertal development in adolescent female rats

**DOI:** 10.1186/s13020-017-0156-7

**Published:** 2018-01-12

**Authors:** Sun Haeng Lee, Hyun Jeong Lee, Sung Hyun Lee, Young-Sik Kim, Donghun Lee, Jiu Chun, Jin Yong Lee, Hocheol Kim, Gyu Tae Chang

**Affiliations:** 1 Department of Clinical Korean Medicine, Graduate School, Kyung Hee University, Seoul, 02447 Republic of Korea; 2 Department of Pediatrics of Korean Medicine, Kyung Hee University Korean Medicine Hospital, Kyung Hee University Medical Center, Seoul, 02447 Republic of Korea; 3 Department of Herbal Pharmacology, College of Korean Medicine, Kyung Hee University, Seoul, 02447 Republic of Korea; 4 Korea Institute of Science and Technology for Eastern Medicine (KISTEM), NeuMed Inc., Seoul, 02440 Republic of Korea; 5 Department of Pediatrics of Korean Medicine, Kyung Hee University Hospital at Gangdong, Dongnam-ro 892, Gangdong-gu, Seoul, 05278 Republic of Korea

**Keywords:** Huang Bai, Bone growth, Insulin-like growth factor-1, Bone morphogenetic protein-2, Vaginal opening, Ovarian weight, Uterine weight

## Abstract

**Background:**

To evaluate the effects of Huang Bai (*Phellodendron amurense*) on growth and maturation in adolescent female rats.

**Methods:**

Female Sprague–Dawley rats (28 days old; n = 72) were divided into six daily treatment groups: control (distilled water), Huang Bai (100 and 300 mg/kg), recombinant human GH (rhGH; 20 μg/kg), estradiol (1 μg/kg), and triptorelin (100 μg). Body weight, food intake, and vaginal opening were measured daily from postnatal day (PND) 28 to PND 43. Tetracycline (20 mg/kg) was injected on PND 41. After sacrifice on PND 43, the ovaries and uterus were weighed, and the tibias were fixed in 4% paraformaldehyde. Decalcified and dehydrated tibias were sectioned at a thickness of 40 μm, and sectioned tissues were examined with a fluorescence microscope. Insulin-like growth factor (IGF)-1 and bone morphogenetic protein (BMP)-2 were detected using immunohistochemistry.

**Results:**

Relative to controls, body weight was higher in the triptorelin group. Bone growth rate increased in the Huang Bai 100 mg/kg (354.00 ± 31.1 μm/day), rhGH (367.10 ± 27.11 μm/day), and triptorelin (374.50 ± 25.37 μm/day) groups. Expression of IGF-1 and BMP-2 in the hypertrophic zone was higher in all experimental groups. Vaginal opening occurred earlier in the estradiol group (PND 33.58 ± 1.62) than in controls and later in the triptorelin group (PND > 43). Ovarian and uterine weights were lower in the oestradiol and triptorelin groups. However, Huang Bai had nonsignificant effects on vaginal opening and the weights of ovaries and the uterus.

**Conclusions:**

Huang Bai stimulated bone growth by upregulating IGF-1 and BMP-2 in the growth plate. However, it had no effect on pubertal development.

**Electronic supplementary material:**

The online version of this article (10.1186/s13020-017-0156-7) contains supplementary material, which is available to authorized users.

## Background

Longitudinal bone growth occurs through elongation of the growth plate, a cartilage layer between the epiphysis and metaphysis [1, 2]. The growth plate consists of three distinct zones: the resting, proliferative, and hypertrophic zones. Chondrocytes in the growth plate are differentiated from the top of the resting zone to the bottom of the hypertrophic zone, and the middle proliferative zone is the major location for chondrocyte proliferation for bone elongation [2, 3]. Growth and differentiation of the growth plate are regulated by growth hormone (GH) and cell-signalling polypeptides such as insulin-like growth factor (IGF)-1 and bone morphogenetic protein (BMP)-2 [4]. The bone growth rate and expression of IGF-1 and BMP-2 are useful indicators of growth.

Reproductive maturation requires activation of the hypothalamic–pituitary–gonadal (HPG) axis. Pulsatile gonadotropin-releasing hormone (GnRH) from the hypothalamus stimulates luteinizing hormone (LH) and follicle-stimulating hormone (FSH) release from the pituitary [5]. LH and FSH stimulate gonadal growth and maturation for the biosynthesis of gametes and sex hormones [6]. Pubertal onset in female rats has been traditionally identified by estrogen-mediated vaginal opening, observed as a visible hole in the membranous coating of the vaginal orifice [7]. In addition, uterotropic assays including ovarian and uterine weight are considered gold standards for identifying estrogenic activity in rats [8]. Vaginal opening and the weight of ovaries and uterus are helpful indicators of pubertal development.

Huang Bai has been used to treat diarrhoea, jaundice, leucorrhoea, stranguria, and swelling of the knee and foot by clearing heat and drying dampness; sores, burns, and eczema by purging fire and detoxifying; and fever by clearing deficiency heat [9]. In our previous in vitro study, Huang Bai promoted GH mRNA and protein in pituitary cells and inhibited GnRH mRNA expression in hypothalamus cells [10]. However, the growth-promoting and maturation-inhibiting effects of Huang Bai were not identified based on that in vivo study.

The present study explored the effects of Huang Bai on growth and pubertal development in adolescent female Sprague–Dawley rats. Huang Bai was compared with recombinant human growth hormone (rhGH; positive control for growth promotion), estradiol (negative control for maturational cessation), and triptorelin (positive control for maturational cessation). The effect on growth was measured based on the daily bone growth rate and expression of BMP-2 and IGF-1 in the tibial growth plate. The effect on pubertal maturation was measured based on vaginal opening and ovarian and uterine weight.

## Methods

The minimum standards of reporting checklist (Additional file 1) contains details of the experimental design, statistics, and resources used in this study.

### Sample preparation

The cortex of *Phellodendron amurense* (Huang Bai) was imported from Sichuan, China (Kyung Hee Herb Pharm.; Gangwon, Republic of Korea). A total of 400 g of dried Huang Bai was extracted with 4000 mL of 100 °C distilled water (DW) for 3 h with a reflux heater. The extracted fluid was filtered with filter paper (Hyundai Micro Co.; Seoul, Republic of Korea), after which the filtered fluid was evaporated to a volume < 2000 mL using a rotary evaporator (Sunileyela Co.; Gyeonggi, Republic of Korea), and lyophilised using a freeze-dryer (OperonTM; Seoul, Republic of Korea). The powder was stored at − 20 °C. The yield of freeze-dried Huang Bai was approximately 10.4%.

**Fig. 1:**
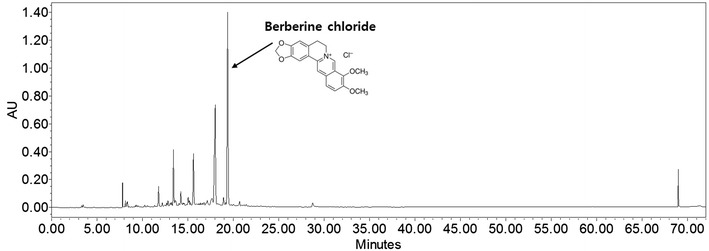
High-performance liquid chromatography of Huang Bai

The quantitative authentication of Huang Bai was performed using a Waters instrument (Milford, MA, USA) equipped with a Waters 1525 pump, Waters 2707 autosampler, and a Waters 2998 PDA detector with a Sunfire™ Octadecyl silyl silica C18 column (particle size, 5 μm; 250 × 4.6 mm). The column was equilibrated with 0.1% phosphoric acid (solvent A) and acetonitrile (solvent B) at a flow rate of 1.0 mL/min. The column was eluted as follows: 0–60 min, 0% solvent B; 60–67 min, 100% solvent B; 67–72 min, 0% solvent B. The high-performance liquid chromatogram of Huang Bai is shown in Fig. 1. Huang Bai contained one representative component: 24.36 mg/g berberine chloride.

### Animals

A total of 72 intact 21-day-old female Sprague–Dawley rats were purchased from Samtako Co. (Gyeonggi, Republic of Korea). The sample size was based on recent experiments conducted in our laboratory [11]. The rats were divided into four body weight groups (30–40, 40–50, 50–60, and 60–70 g). They were marked on the tail and housed four per cage under controlled temperature (23 ± 2 °C), humidity (55 ± 10%), and lighting (lights on from 7 a.m. to 7 p.m.) with free access to food and water intake. After 1 week of acclimatisation, the 28-day-old rats, weighing 75 ± 10 g, were administered their respective treatments. All experimental procedures were performed according to the animal care guidelines of Kyung Hee University’s Institutional Animal Care and Use Committee [Protocol Number KHUASP(SE)-15-052].

### Treatments

The 18 cages were randomly allocated into six groups (three cages per group) according to the treatment regimen. The DW (12 rats) and Huang Bai (100 and 300 mg/kg; 12 rats each) groups were orally administered twice daily at 7 a.m. and 7 p.m. at 10.0 mL/kg. The rhGH (20 μg/kg; 12 rats) (LG Life Science; Seoul, Republic of Korea) [12] and 17β-estradiol (1 μg/kg; 12 rats) (Sigma-Aldrich; MO, USA) [13] groups were administered subcutaneous injections once daily at 7 a.m. at 1.0 mL/kg. A fixed dose and volume of triptorelin (100 μg/0.4 mL; 12 rats) (Ferring AG; Baarermatte, Switzerland) was intraperitoneally injected once daily at 7 a.m. [14].

Body weights of animals, food intake per cage, and vaginal opening were measured daily, and treatments were continued from postnatal day (PND) 28 to PND 43. On PND 41, all animals received an intraperitoneal injection of tetracycline hydrochloride (20 mg/kg, Sigma-Aldrich) in 5.0 mL/kg saline for the fluorescent dye under ultraviolet illumination. On PND 43, all rats were sacrificed under anaesthesia. The ovaries and uterus were dissected with the cervix attached, and trimmed free of fat. The wet weights were then obtained. The tibias were dissected free of the soft tissue, and the bones were immediately fixed in 4% paraformaldehyde.

### Measurement of longitudinal bone growth rate

Fixed tibias were decalcified by immersion in 50 mM ethylene diamine tetra acetic acid solution (Sigma-Aldrich) for 2 d. Decalcified bones were dehydrated by immersion in 30% sucrose (Sigma-Aldrich) for 1 day. Dehydrated bones were sectioned longitudinally at a thickness of 40 μm with a sliding microtome (Leica CM1860; Berlin, Germany). Sections of bone tissue were mounted on gelatinised glass slides and photographed with a fluorescence microscope (Olympus; Tokyo, Japan). The longitudinal bone length between the fluorescent line formed by tetracycline and the epiphyseal end line of the growth plate was measured by two blinded investigators (SHL and YSK) using Image J software (National Institutes of Health; MD, USA). The longitudinal bone growth rate was calculated as the measured length divided by the time between tetracycline injection and death.

### Immunohistochemistry for expression of IGF-1 and BMP-2

Dehydrated tibia sections were rinsed twice in 0.1 M phosphate buffer saline (PBS) for 15 min and incubated with 1% triton X-100 (Sigma-Aldrich) and 0.5% bovine serum albumin (BSA, Sigma-Aldrich) mixed in PBS for 10 min at room temperature. The samples were then washed twice in PBS/BSA for 15 min and incubated with 1:200 rabbit IGF-1 primary antibody or 1:200 goat BMP-2 primary antibody (Santa Cruz Biotechnology; TX, USA) overnight at room temperature in a humid chamber. After 24 h, sections were washed twice in PBS/BSA for 15 min and incubated with 1:200 biotinylated anti-goat secondary antibody (Vector Laboratories; CA, USA) or 1:200 biotinylated anti-rabbit secondary antibody (Jackson Immuno Research Laboratories; PA, USA) for 60 min. After being washed twice with PBS/BSA for 15 min, the sections were incubated with 1:100 avidin-biotinperoxidase complex (Vectastain ABC Kit; Vector Laboratories) for 60 min at room temperature. After two washings with 0.1 M phosphate buffer for 15 min, the sections were stained with 0.05% 3,3-diaminobenzidine solution containing hydrogen peroxidase in PBS. Samples were checked for suitable staining with a microscope, and then the reaction was stopped by washing with PBS for 5 min. The samples were then dehydrated with 50, 75, 95, and 100% ethanol and xylene in order. Dehydrated sections were mounted on glass slides with permount medium solution (Fisher Scientific; NJ, USA). When the sections fell off the slides, we immediately reattached them using soft brushes. Finally, the sections were photographed with a microscope. The percentage of labelled chondrocytes was calculated by counting stained and total chondrocytes in two parallel columns using Image J software [3].

### Statistical analysis

Data are expressed as the mean ± standard deviation (SD) and were analysed using the Student’s t-test (GraphPad Software, Inc.; CA, USA). P-values < 0.05 were considered statistically significant.

## Results

### Body weight and food intake

From PND 28 to PND 43, body weight gains and food intake of the control, Huang Bai 100 and 300 mg/kg, rhGH, 17β-estradiol, and triptorelin groups were compared (Table 1). There were no statistically significant differences in body weight between any treatment group and the control group (*P* > 0.05), except the triptorelin group (*P* < 0.001). There were no statistically significant differences in food intake between any treatment group and the control group (*P* > 0.05).

**Table 1:** Body weight and daily food intake gains (g) for 15 days in female adolescent rats

	Control	HB100	HB300	rhGH	Estradiol	Triptorelin
BW gain	66.96 ± 4.45	65.04 ± 7.14	67.18 ± 10.91	72.25 ± 8.34	64.04 ± 8.91	82.46 ± 5.52
P-value	–	0.44	0.95	0.07	0.32	< 0.001***
DFI gain	13.50 ± 5.27	14.83 ± 2.75	13.25 ± 0.35	16.17 ± 7.11	17.17 ± 1.76	28.33 ± 7.97
P-value	–	0.72	0.95	0.63	0.32	0.05

*Control* DW, *HB100* Huang Bai (100 mg/kg), *HB300* Huang Bai (300 mg/kg), *rhGH* recombinant human growth hormone (20 μg/kg), *Estradiol* 17β-estradiol (1 μg/kg), *Triptorelin* triptorelin (100 μg), *BW* body weight, *DFI* daily food intake

Each value is the mean ± SD of 12 rats except HB300 (11 rats). *** *P* < 0.001 compared to controls

### Longitudinal bone growth rate

**Fig. 2:**
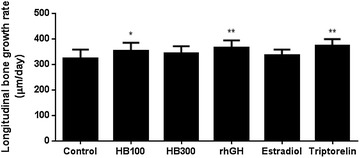
The daily longitudinal bone growth rate was calculated from the 2-day longitudinal growth plate length. Each value is shown as the mean ± SD for 12 rats, except HB300 (11 rats). Statistical analysis: *P < 0.05, **P < 0.01 compared to control (DW–administrated group). HB100: Huang Bai (100 mg/kg, p.o.), HB300: Huang Bai (300 mg/kg, p.o.), rhGH: recombinant human growth hormone (20 μg/kg, s.c.), estradiol: 17β-estradiol (1 μg/kg, s.c.), triptorelin: triptorelin (100 μg, i.p.)

The longitudinal bone growth rate of the control, Huang Bai 100 and 300 mg/kg, rhGH, estradiol, and triptorelin groups were 323.80 ± 34.55, 354.00 ± 31.1 μm/day (*P* = 0.043), 342.60 ± 28.91 μm/day (*P* = 0.195), 367.10 ± 27.11 μm/day (*P* = 0.005), 335.10 ± 23.12 μm/day (*P* = 0.360), and 374.50 ± 25.37 μm/day (*P* = 0.002), respectively. Longitudinal bone growth rates of the Huang Bai 100 mg/kg, rhGH, and triptorelin groups were significantly higher than that of the control group (Fig. 2).

### Expression of IGF-1 and BMP-2

**Fig. 3:**
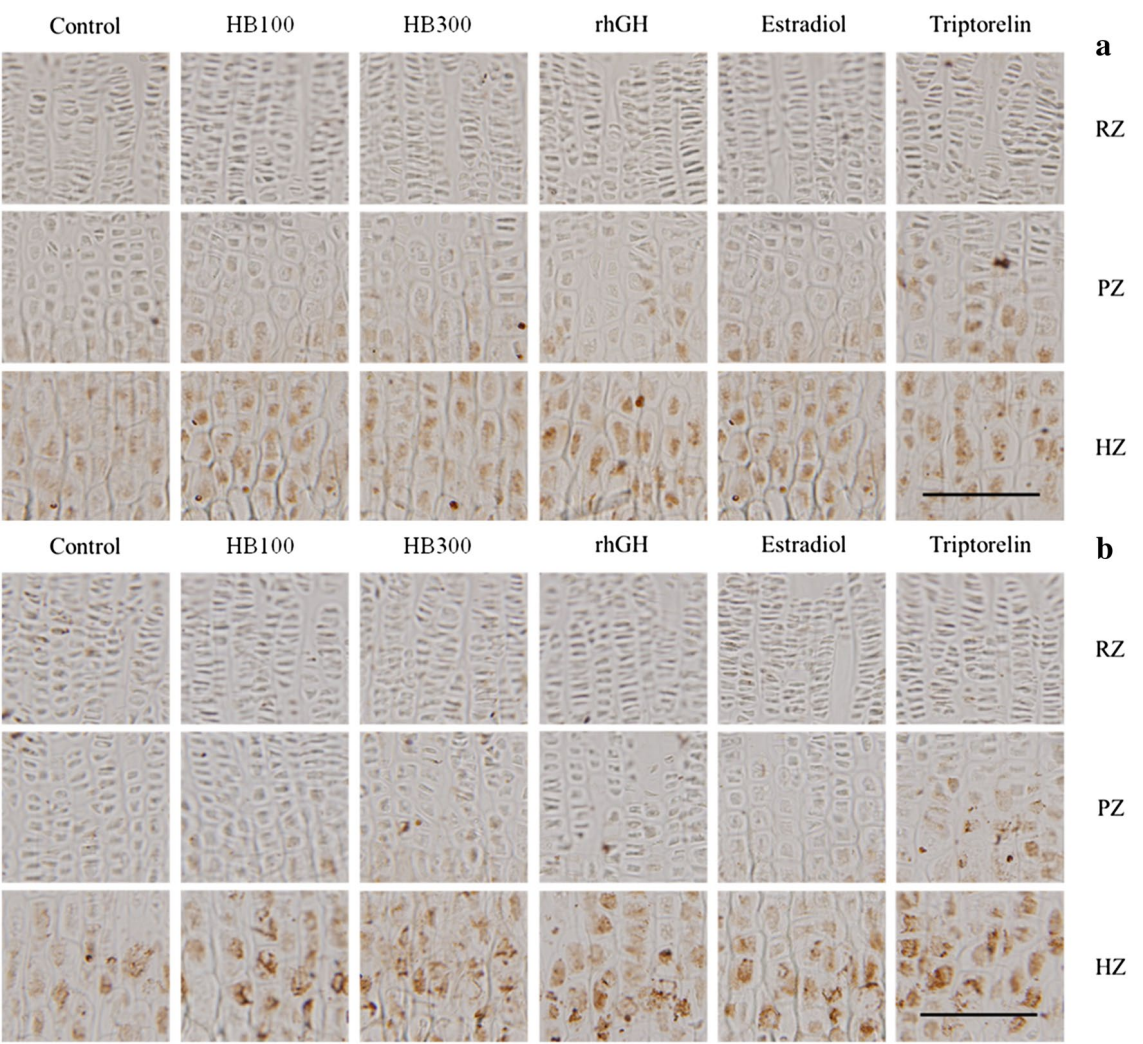
IGF-1 **a** and BMP-2 **b** in the growth plate was detected using immunohistochemistry. Control: DW, HB100: Huang Bai (100 mg/kg, p.o.), HB300: Huang Bai (300 mg/kg, p.o.), rhGH: recombinant human growth hormone (20 μg/kg, s.c.), estradiol: 17β-estradiol (1 μg/kg, s.c.), triptorelin: triptorelin (100 μg, i.p.). RZ: resting zone, PZ: proliferative zone, HZ: hypertrophic zone. Scale bar = 100 μm

**Fig. 4:**
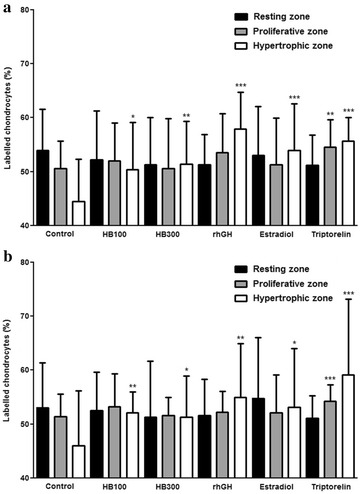
Percentage of IGF-1 **a** and BMP-2 **b** labelled chondrocytes was calculated by counting stained and total chondrocytes in two parallel columns. Each value is shown as the mean ± SD of 12 rats, except HB300 (11 rats). Statistical analysis: *P < 0.05, **P < 0.01, ***P < 0.001 compared to control (DW– administrated group). HB100: Huang Bai (100 mg/kg, p.o.), HB300: Huang Bai (300 mg/kg, p.o.), rhGH: recombinant human growth hormone (20 μg/kg, s.c.), estradiol: 17β-estradiol (1 μg/kg, s.c.), triptorelin: triptorelin (100 μg, i.p.)

IGF-1 and BMP-2 in the resting, proliferative, and hypertrophic zones of the tibial growth plate were detected using immunohistochemical methods. IGF-1 and BMP-2 showed higher levels of expression in the hypertrophic zone in all experimental groups than in that of the control group. The triptorelin group showed a slightly higher expression level of IGF-1 and BMP-2 in the proliferative zone compared with the control group. Huang Bai had higher expression levels of IGF-1 and BMP-2 than the control, but lower levels than the rhGH, estradiol and triptorelin groups (Figs. 3, 4).

### Vaginal opening

**Fig. 5:**
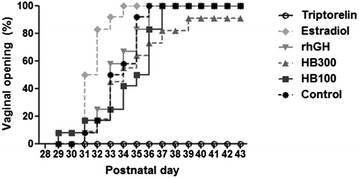
Percentage of female rats with vaginal opening occurring between PND 28 and 43. One rat in the Huang Bai 300 mg/kg group and all rats in the triptorelin group failed to show vaginal opening. Control: DW (p.o.), HB100: Huang Bai (100 mg/kg, p.o.), HB300: Huang Bai (300 mg/kg, p.o.), rhGH: recombinant human growth hormone (20 μg/kg, s.c.), estradiol: 17β-estradiol (1 μg/kg, s.c.), triptorelin: triptorelin (100 μg, i.p.)

The percent incidence of vaginal opening in each group is shown in Fig. 5. All rats in the triptorelin group and one rat in the Huang Bai 300 mg/kg group did not show vaginal opening, whereas all rats of the control, Huang Bai 100 mg/kg, rhGH, and estradiol groups showed vaginal opening prior to sacrifice. The vaginal opening days of the control, Huang Bai 100 and 300 mg/kg, rhGH, estradiol, and triptorelin groups were 34.17 ± 1.95, 34.50 ± 2.47 (*P* = 0.717), > 35.09 ± 3.51 (no vaginal opening in one rat), 33.58 ± 1.62 (*P* = 0.434), 31.58 ± 1.24 (*P* < 0.001), and > 43 (no vaginal opening in any rat), respectively. The estradiol group showed significantly earlier vaginal opening, and the vaginal opening days of the Huang Bai and rhGH groups were not significantly different from that of the control group.

### Ovarian and uterine weight

The ovarian weights and indices of the control, Huang Bai 100 and 300 mg/kg, rhGH, estradiol, and triptorelin groups are described in Table 2. Ovarian indices were calculated as ovarian weight (g) per kg body weight. Ovarian weights of the estradiol and triptorelin groups were significantly lower than those of the control group (*P* < 0.001).

**Table 2:** Ovarian weight (g) and index (g/kg) at postnatal day 43 in female adolescent rats

	Control	HB100	HB300	rhGH	Estradiol	Triptorelin
Ovarian weight	0.10 ± 0.01	0.10 ± 0.03	0.09 ± 0.02	0.09 ± 0.02	0.07 ± 0.01	0.03 ± 0.01
P-value	–	1.000	0.612	0.431	< 0.001***	< 0.001***
Ovarian index	0.66 ± 0.08	0.69 ± 0.24	0.64 ± 0.16	0.61 ± 0.15	0.54 ± 0.11	0.17 ± 0.08
P-value	–	0.791	0.659	0.300	0.004**	< 0.001***

*Control* DW, *HB100* Huang Bai (100 mg/kg), *HB300* Huang Bai (300 mg/kg), *rhGH* recombinant human growth hormone (20 μg/kg), *Estradiol* 17β-estradiol (1 μg/kg), *Triptorelin* triptorelin (100 μg)

Each value is the mean ± SD of 12 rats except HB300 (11 rats). ** *P* < 0.01, *** *P* < 0.001 compared to controls

The uterine weights and indices of each group are described in Table 3. Uterine indices were calculated as uterine weight (g) per body weight (kg). Uterine weights of the estradiol (P < 0.01) and triptorelin (P < 0.001) groups were also significantly lower than those of the control group.

**Table 3:** Uterine weight (g) and index (g/kg) at postnatal day 43 in female adolescent rats

	Control	HB100	HB300	rhGH	Estradiol	Triptorelin
Uterine weight	0.39 ± 0.09	0.37 ± 0.14	0.38 ± 0.17	0.39 ± 0.12	0.29 ± 0.05	0.06 ± 0.02
P-value	–	0.663	0.838	0.923	0.002**	< 0.001***
Uterine index	2.76 ± 0.61	2.70 ± 1.04	2.69 ± 1.14	2.67 ± 0.80	2.13 ± 0.42	0.41 ± 0.13
P-value	–	0.863	0.857	0.785	0.008**	< 0.001***

*Control* DW, *HB100* Huang Bai (100 mg/kg), *HB300* Huang Bai (300 mg/kg), *rhGH* recombinant human growth hormone (20 μg/kg), *Estradiol* 17β-estradiol (1 μg/kg), *Triptorelin* triptorelin (100 μg)

Each value is the mean ± SD of 12 rats except HB300 (11 rats). ** *P* < 0.01, *** *P* < 0.001 compared to controls

**Fig. 6:**
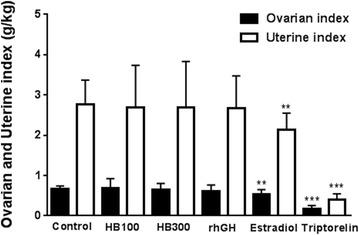
Ovarian and uterine indices after sacrifice were calculated as ovarian and uterine weight (g) per body weight (kg). Each value is shown as the mean ± SD of 12 rats, except HB300 (11 rats). Statistical analysis: **P < 0.01, ***P < 0.001 compared to control (DW– administrated group).* HB100* Huang Bai (100 mg/kg, p.o.),* HB300* Huang Bai (300 mg/kg, p.o.),* rhGH* recombinant human growth hormone (20 μg/kg, s.c.),* estradiol* 17β-estradiol (1 μg/kg, s.c.),* triptorelin* triptorelin (100 μg, i.p.)

The ovarian and uterine indices of both the estradiol group and the triptorelin group were significantly lower than those of the control group, with *P* < 0.01 and *P* < 0.001, respectively (Fig. 6).

## Discussion

Sprague–Dawley rats are commonly used in growth and reproductive experiments because of their outbred genetic background and the availability of a large volume of historical health data for many age points [15]. In this study, the effects of 100 and 300 mg/kg Huang Bai on growth and pubertal maturation were compared with those of rhGH, estradiol, and triptorelin. These two concentrations of Huang Bai were converted from the conventional daily dose of 0.5 and 1.5 g in 30 kg children. The rhGH stimulates bone growth and weight gain in a dose-dependent manner in rats [16]. Estradiol increases wet weight, volume, and the uterine epithelium in immature female rats [17]. Triptorelin upregulates pituitary GnRH receptors and serum gonadotropins in low doses, while inhibiting GnRH receptors and gonadotropins at high doses in rats [18]. In this experiment, a rat in the Huang Bai 300 mg/kg group showed body weight decrease after PND 40 due to intestinal obstruction (identified post-mortem). Therefore, the rat was excluded from our analyses.

Huang Bai did not stimulate weight gain; most daily weights and food intake levels were not significantly different from the control. However, triptorelin did stimulate weight gain. This may be an adverse event of GnRH agonists, which partially cause adiposity [19] and excessive weight gain [20].

The 100 mg/kg of Huang Bai, rhGH, and triptorelin significantly increased the bone growth rate, by 9.3, 13.4, and 15.7%, respectively, compared to the control. However, 300 mg/kg of Huang Bai had a lower growth effect than 100 mg/kg. Huang Bai has a bitter taste, and the theory of “bitter/cold medicines damage the spleen and stomach” suggests that this bitter-tasting herb may cause anorexia, dyspepsia, or gastric motility disorder. In a previous study, Huang Bai decreased the secretion of gastric juice, increased the pH of gastric juice, and decreased activation of pepsin in rats [21]. In this study, one rat from the 300 mg/kg Huang Bai group had an intestinal obstruction. The lower growth effect of the higher dose Huang Bai may be due to its increased burden on the gastrointestinal tract. Further studies are required to determine the appropriate dose and preparation process for Huang Bai so as to reduce the burden on the gastrointestinal tract. The triptorelin group showed the highest bone growth rate, but this result may have been due to confounding effects of the significant weight gain in this group.

IGF-1 and BMP-2 were highly expressed in the hypertrophic zone following Huang Bai administration, reflecting the proliferation of chondrocytes. IGF-1 regulates cell differentiation, proliferation, and maturation through autocrine and paracrine activity [2], and BMP-2 regulates growth plate chondrogenesis and induces ectopic bone formation and skeletal development [22, 23]. BMP-2 also modulates mitogenic IGF-1 action of chondrocytes in the epiphyseal plate [4]. Huang Bai stimulated chondrocyte proliferation and ectopic bone elongation by increasing IGF-1 and BMP-2 expression in the growth plate. The longitudinal bone growth rate, and IGF-1 and BMP-2 expression in the growth plate indicate that Huang Bai stimulated ectopic skeletal growth by promoting chondrocyte differentiation. This result is in agreement with the GH3 cell study showing that Huang Bai increased GH mRNA and protein expression in pituitary cells [10].

Huang Bai delayed vaginal opening in a dose-dependent manner, but differences from the control were not significant. Further studies using other doses or rats with precocious puberty are required because herbal prescriptions including Huang Bai have been associated with delayed vaginal opening in precocious puberty rats [24–28]. In contrast, vaginal opening day of the estradiol and triptorelin groups was significantly different from that for the control group. The estradiol group showed earlier vaginal opening, and no rats in the triptorelin group showed vaginal opening.

Huang Bai did not affect hypertrophy of the ovaries and uterus, although herbal prescriptions that include Huang Bai have been associated with lower ovarian weight [29] and with lower ovary and uterus indices [30] in intact rats. In contrast, wet weight and ovary and uterus indices were significantly lower in the estradiol and triptorelin groups than in the control group. In a previous study, ovarian and uterine weights of persistent oestrus- or dioestrus-cycle rats were lower than those of rats with normal oestrus cycles [31]. Long-term estradiol or triptorelin administration may change the oestrus cycle of rats from normal to a persistent cycle of oestrus and dioestrus.

Results for vaginal opening and ovary and uterus indices indicate that Huang Bai did not inhibit pubertal maturation in the female rat, although it inhibited GnRH mRNA expression in the hypothalamus [10]. The difference between these in vitro results and multiple studies of herbal mixture may be explained by insufficient gastrointestinal absorption, the blood–brain barrier, or other confounding factors of the HPG axis. Further studies are required to explore these discordances.

## Conclusions

Huang Bai stimulates longitudinal bone growth and chondrocyte proliferation by upregulating BMP-2 and IGF-1 expression in the growth plate. However, it has no effects on pubertal onset and estrogenic activity. Treatment with rhGH, which promotes growth, is generally safe in children but is associated with pseudotumor cerebri [32], slipped capital femoral epiphysis [33], progression of scoliosis [34], and/or the development of GH antibodies [35]. Huang Bai may be an alternative treatment for stimulating bone growth without affecting pubertal process.

## Additional file

**Additional file 1.** Minimum standards of reporting checklist.

## Abbreviations

BMP: bone morphogenetic protein
BSA: bovine serum albumin
FSH: follicle-stimulating hormone
GH: growth hormone
GnRH: gonadotropin-releasing hormone
HPG: hypothalamic–pituitary–gonadal
IGF: insulin-like growth factor
LH: luteinizing hormone
PBS: phosphate-buffered saline
PND: postnatal day
rhGH: recombinant human growth hormone
SD: standard deviation
